# Quantum Chemical Properties of Fluorouracil/XZn_11_O_12_ (X = Zn, Cu, Fe, Ni) Nanocomplexes and Interactions with Human Serum Albumin

**DOI:** 10.1002/open.202500535

**Published:** 2026-03-04

**Authors:** Mohan Bahadur Kshetri, Navin Sharma, Kamal Khanal, Madhav Prasad Ghimire, Tika Ram Lamichhane

**Affiliations:** ^1^ Central Department of Physics Tribhuvan University Kirtipur Nepal

**Keywords:** density functional theory, fluorouracil, molecular docking, transition metal‐doped Zn_12_O_12_ nanocage

## Abstract

This research focused on structural, electronic, and interaction properties of fluorouracil (5‐FU) adsorbed on transition metal (TM)‐doped ZnO nanoclusters (XZn_11_O_12_, where X = Zn, Cu, Fe, Ni) using density functional theory (DFT) at the B3LYP/LANL2DZ level of calculations in the gas phase. Among the studied nanocomplexes, 5‐FU@NiZn_11_O_12_ exhibited the highest dipole moment (8.08 D), indicating strong polarization and potential surface reactivity though it has a less negative adsorption energy (−20.97 kcal/mol) compared to 5‐FU@FeZn_11_O_12_ (−35.51 kcal/mol) and 5‐FU@CuZn_11_O_12_ (−28.69 kcal/mol). TM doping significantly reduced the highest occupied molecular orbital–lowest unoccupied molecular orbital gap, with 5‐FU@NiZn_11_O_12_ showing the lowest value (2.44 eV), followed by 5‐FU@FeZn_11_O_12_ (2.53 eV) and 5‐FU@CuZn_11_O_12_ (3.18 eV), suggesting enhanced charge transfer and chemical reactivity. The results from molecular electrostatic potential, quantum theory of atoms in molecules, and non–covalent interaction/reduced density gradient analyses were also in favor of Ni‐doped ZnO nanocomplex. Based on the DFT results, 5FU@NiZn_11_O_12_ was selected to analyze its interactions with human serum albumin (HSA). From molecular docking of 5‐FU@NiZn_11_O_12_, binding energy (−5.36 kcal/mol) and inhibition constant (117.15 μM) exhibited stronger interactions with HSA, so that it acts as a potential candidate of drug delivery system for anticancer therapy. However, these predictive insights require further experimental validation.

## Graphical abstract

Adsorption of fluorouracil (5‐FU) on XZn11O12 (X = Zn, Cu, Fe, Ni) nanoclusters and its interactions with human serum albumin (HSA) have been studied using density functional theory and molecular docking. Reduced HOMO‐LUMO gap of 5‐FU@NiZn11O12 nanocomplex suggests enhanced charge transfer activity. The favorable binding interactions of 5‐FU@NiZn11O12 with HSA exhibit its potential for drug delivery and anticancer therapy.

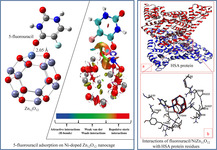

## 1 Introduction

Annually resulting in millions of deaths, cancer, fundamentally driven by uncontrolled cell growth and proliferation, stands as a paramount global health crisis, with intensifying socio‐medical burdens [1]. In response to this formidable adversary, chemotherapy has emerged as a cornerstone of oncology, currently standing at the forefront of systemic treatment strategies [2]. Fluorouracil (5‐FU), a widely used chemotherapy drug, especially for colorectal, breast, head and neck, and gastrointestinal cancers, is a pyrimidine analog that combats cancer by blocking thymidylate synthase (TS) and inserting its active forms into RNA and DNA, thereby disrupting their normal functions [3]. Despite its therapeutic power, 5‐FU is constrained by a short biological half‐life and notable bone marrow toxicity [4], along with risks of cardiotoxicity [5] and various adverse effects, including gastrointestinal ulceration, respiratory distress, hematologic complications, neurotoxicity, and possible fatality [6]. To address these limitations, nanostructure‐based drug systems have gained attention as a novel approach, offering unique architectures such as nanosheets, nanotubes, or nanocages, thereby improving drug solubility, minimizing toxicity and side effects, and facilitating site‐specific drug targeting [4].

The metal oxide nanostructures exhibit several advantageous features in cancer therapy, especially when functionalized or adsorbed with anticancer drugs, as reported in the literature [7, 8, 9]. Transition metal (TM)‐doped nanostructures have recently gained significant attention, as doping can further enhance their physicochemical and therapeutic properties. For instance, Arshad et al. [10] reported that Ni‐ and Zn‐doped Mg_12_O_12_ nanoclusters exhibit strong adsorption energies, reduced band gaps, and stable electronic properties, indicating their potential as drug carriers for metformin. Similarly, Ogunwale et al. [4] explored pristine and functionalized Ca_12_O_12_ nanocages for 5‐FU loading and found strong interactions in both the unmodified and—NH_2_‐functionalized systems. Beyond oxide materials, Rezaei‐Sameti et al. [11] investigated pristine and Sc/Ti‐doped B_12_P_12_ nanocages under static electric fields and found improved 5‐FU binding with increasing field strength, highlighting the importance of external stimuli and dopants. Zhihong et al. [12] performed a density functional theory (DFT) study on 5‐FU interaction with pristine and Fe‐, Mg‐, Al‐, and Ga‐doped Zn_12_O_12_ nanoclusters and reported enhanced adsorption energies and reactivity upon doping, especially with Mg and Ga. Mohammed et al. [13] studied 5‐FU adsorption on pristine and metal‐doped ZnO nanosheets, revealing significant changes in electronic properties upon doping with Cu, Au, and Ag, and stronger drug–surface interaction compared to the undoped sheet. These studies collectively emphasize the crucial role of surface modification and electronic tuning in improving 5‐FU adsorption, sensing, and delivery. In the present study, we investigated the quantum chemical properties of fluorouracil (5‐FU)‐loaded XZn_11_O_12_ (X = Zn, Cu, Fe, Ni) nanocomplexes. Herein, zinc oxide (ZnO) nanocages were chosen as the base matrix, since they have attracted widespread interest in biomedical research owing to their excellent biocompatibility, low toxicity, thermal stability, cost‐effectiveness, and favorable biosafety profile [14].

Human serum albumin (HSA), the primary protein in human plasma, plays a crucial role in the transport, distribution, and metabolism of various endogenous and exogenous substances, including pharmaceutical compounds [15]. Hassanian et al. [16] reported that ZnO nanoparticles interact with HSA mainly through electrostatic interactions between Zn/O atoms and acidic or basic residues, which could result in its conformational changes. Similarly, Mohamed et al. [17] demonstrated that 5‐FU exhibits relatively low binding affinity and specificity toward HSA compared with other anticancer drugs. Furthermore, Chinnathambi et al. [18] observed that both azapropazone and 5‐FU preferentially bind to site I rather than site II of BSA (which shares ∼76% homology with HSA), with comparable docking scores and hydrogen‐bond interactions involving hydrophobic residues.

Previous experimental studies have also demonstrated the enhanced therapeutic performance of drug‐loaded ZnO nanostructures. For instance, Al‐Ajmi et al. [19] explored that 5‐FU encapsulated ZnO nanoparticles offer higher therapeutic potential against the MCF‐7 breast cancer cell line compared to free 5‐FU, while Kim et al. [20] showed that doxorubicin‐wrapped ZnO nanoclusters exhibit promising activity against colorectal cancer. Beyond ZnO‐based systems, Al‐Jorani et al. [21] employed DFT and molecular docking to demonstrate the anticancer potential of tetrasubstituted imidazole derivatives containing a benzothiazole moiety. Although numerous recent studies have examined the adsorption of 5‐FU on pristine or metal‐doped ZnO and other nanocages/nanostructures, as well as the interaction of 5‐FU with HSA, the combined investigation of 5‐FU‐loaded TM‐doped ZnO nanocages and their interaction with HSA remains unexplored. In this study, we systematically investigated the binding affinity, electronic properties, and interaction mechanisms of 5‐FU/XZn_11_O_12_ (X = Zn, Cu, Fe, Ni) nanocomplexes using DFT and molecular docking methods, thereby providing new insights into drug–carrier interactions.

## 2 Methodology

### 2.1 DFT Study

This study employed a Zn_12_O_12_ nanocluster as a representative nanocage model to investigate its interaction with the anticancer drug fluorouracil (5‐FU). Furthermore, to assess the effect of TM doping, one Zn atom was substituted with Cu, Fe, or Ni, forming TM‐doped clusters (XZn_11_O_12_, where X = Zn, Cu, Fe, or Ni). DFT calculations were carried out using the Gaussian 16W software package [22]. All geometry optimizations and energy calculations were performed on the pristine and TM‐doped Zn_12_O_12_ nanocages, as well as their complexes with the anticancer drug 5‐FU, utilizing the B3LYP functional in combination with the LANL2DZ basis set in the Gaussian 16W program. The B3LYP functional was selected due to its proven reliability and computational efficiency in modeling nanostructured materials [23, 24], whereas the LANL2DZ basis set was chosen for its suitability in treating TM atoms efficiently [25]. Although higher‐level functionals and larger basis sets could improve quantitative accuracy, they would significantly increase computational cost, making B3LYP/LANL2DZ a practical compromise for these nanoclusters. Furthermore, this computational approach has been widely adopted in prior studies, as it provides a reliable balance between computational accuracy and efficiency, particularly for large nanocluster systems. Additionally, this study focuses on identifying relative trends rather than exact experimental values.

The density of states (DOS) of the nanocages, 5‐FU molecule, and their corresponding complexes was computed using the GaussSum 3.0 program [26]. Dipole moment and chemical reactivity descriptors were also evaluated at the B3LYP/LANL2DZ level. Additionally, time‐dependent DFT, available in Gaussian 16W, was employed to simulate the UV–visible absorption spectra [27]. Visualization and analysis of molecular orbitals, charge distributions, and spectra were performed using GaussView 6.0 [28]. Furthermore, molecular electrostatic potential (MEP) mapping and Mulliken charge distribution analyses were conducted to assess charge localization and identify potential interaction sites within the complexes [29]. In addition, Multiwfn software [30], in combination with visual molecular dynamics (VMD) software [31], was employed to perform quantum theory of atoms in molecules (QTAIM) analysis and visualize NCI/RDG surfaces, thereby offering deeper insights into the noncovalent interactions (NCI) and electron density topology of the studied systems.

The proposed 5‐FU/XZn_11_O_12_ (X = Zn, Cu, Fe, Ni) nanocomplexes were optimized using convergence criteria: maximum force = 4.5 ×  10^−4^ Hartree/Bohr, RMS force = 3.0 × 10^−4^ Hartree/Bohr, maximum displacement = 1.8 × 10^−3^ Bohr, and RMS displacement = 1.2 ×  10^−3^ Bohr. Moreover, frequency calculations were performed to confirm the absence of imaginary frequencies, ensuring that the optimized geometries correspond to true local minima.

The adsorption energy $\left(E_{\mathrm{ad}}\right)$ of 5‐FU on each nanocage surface was calculated using Equation (1).

(1) $$E_{\mathrm{ad}}=E_{\mathrm{FU}/\text{nanocage}}-(E_{\text{nanocage}}+E_{\mathrm{FU}})$$

where $E_{\mathrm{FU}/\text{nanocage}}$, $E_{\text{nanocage}}$, and $E_{\mathrm{FU}}$ correspond to the energies of the adsorbed complex, Zn_12_O_12_ nanocage, and the 5‐FU molecule, respectively. A negative $E_{\mathrm{ad}}$ value indicates an exothermic process and a strong interaction between the adsorbate and the surface [32].

### 2.2 Molecular Docking Study

The 3D crystal structure of HSA was retrieved from the RCSB Protein Data Bank (PDB ID: 1AO6). The protein structure was refined and prepared for docking using AutoDock Tools (version 4.2.6) [33]. Preparation steps included the removal of crystallographic water molecules, the addition of Kollman charges, and the insertion of polar hydrogen atoms. The 5‐FU molecule, Zn_12_O_12_ nanocage, and the 5‐FU/XZn_11_O_12_ nanocomplexes were optimized using DFT.

For the docking simulations, a grid box of dimensions (60 × 60 × 60 points) was defined, centered at coordinates (27.006, 18.030, and 34.679) with a grid spacing of 0.375 Å. The Lamarckian genetic algorithm was employed using the following parameters: population size of 150, maximum energy evaluations of 2,500,000, and 10 independent docking runs per ligand. All ligands were modeled with zero rotatable bonds to simulate rigid‐body docking.

For the postdocking analysis, the best binding conformers were analyzed using BIOVIA Discovery Studio Visualizer [34], focusing on key NCI including hydrogen bonds, van der Waals forces, hydrophobic contacts, and electrostatic interactions. This molecular docking strategy facilitated evaluation of binding affinity and inhibitory potential of the 5‐FU loaded nanocomplexes in their interaction with HSA. The detailed procedure of implementing DFT and molecular docking to identify structural, electronic, and binding properties of the potential drug candidates was also explained in the recent studies [35, 36, 37].

## 3 Results and Discussion

### 3.1 Structural Properties

**FIGURE 1:**
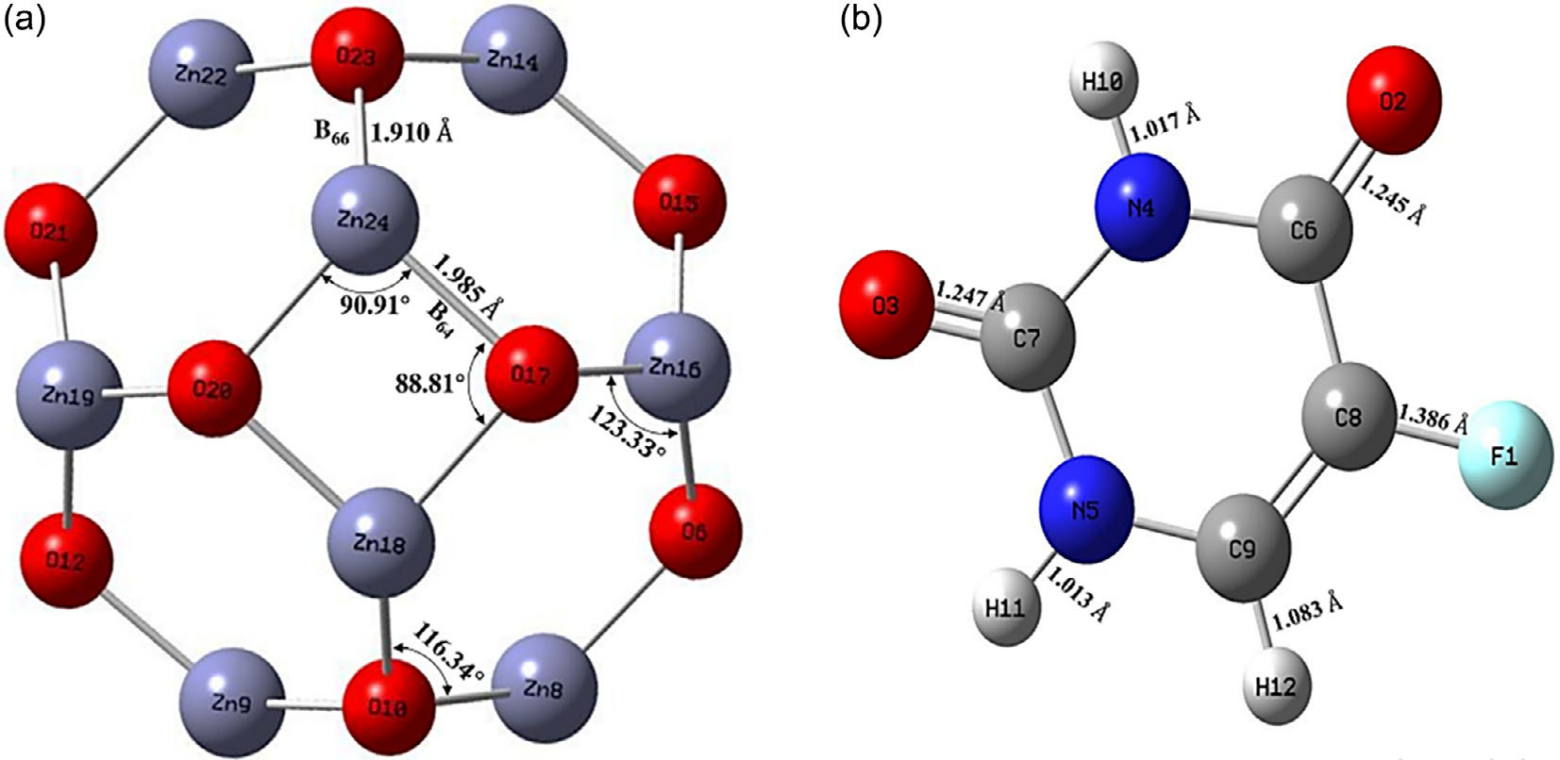
Optimized structures of (a) pristine Zn_12_O_12_ nanocluster with representative Zn–O bond lengths and bond angles, and (b) fluorouracil (5‐FU, PubChem CID: 3385) showing its planar geometry and key bond lengths.

The optimized structure of the pristine Zn_12_O_12_ nanocluster, as shown in Figure 1a, revealed a nearly spherical, cage‐like geometry (T_h_ symmetry), consisting of six tetragonal and eight rings. From a bonding perspective, two distinct types of Zn–O bonds were identified: the B66 bond, two adjacent hexagonal rings, and the B64 bond, which bridges a hexagonal and a tetragonal ring [38, 39]. In our calculations, the B66 bond length was found to be 1.910 Å, while the B64 bond was slightly longer at 1.985Å; both results were in close agreement with earlier theoretical studies [12, 38, 39, 40]. The Zn–O–Zn bond angles within the hexagonal and tetragonal rings were to be 116.34° and 88.81°, respectively, while the corresponding O–Zn–O angles were 123.33° and 90.91°. These geometrical parameters were consistent with those previously reported in literature [32, 41], affirming the structural reliability and accuracy of our optimized model. Furthermore, the optimized structure of 5‐FU (PubChem CID: 3385), as shown in Figure 1b, showed a planar geometry with distinct bond lengths such as F1–C8 (1.386 Å), O3–C7 (1.247 Å), O2–C6 (1.245 Å), N5–H11 (1.013 Å), N4–H10 (1.017 Å), and C9–H12 (1.083 Å). These values aligned closely with earlier DFT/6‐31G* results [42], indicating that our B3LYP/LANL2DZ results were consistent and reliable.

**FIGURE 2:**
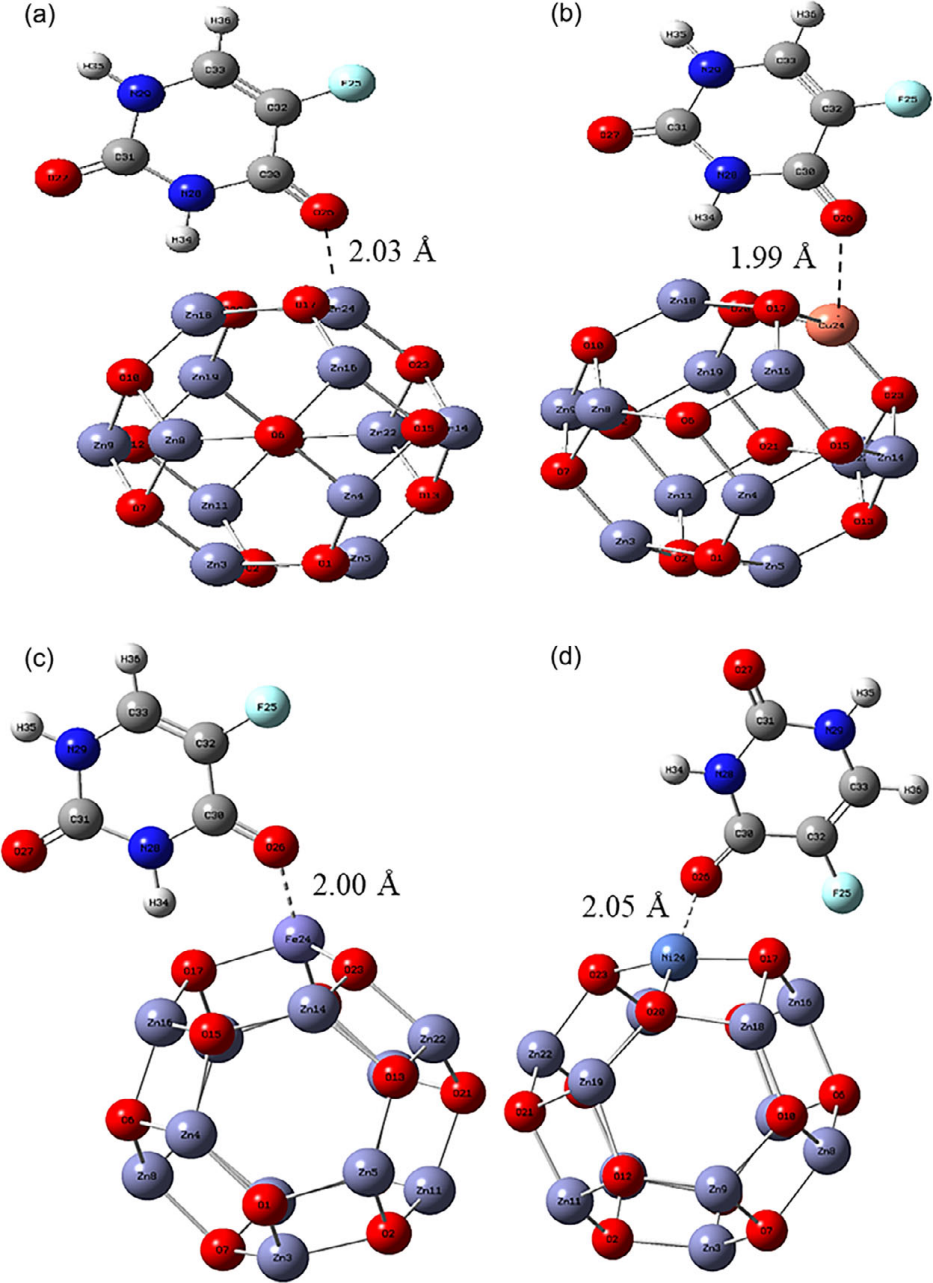
Optimized structures of (a) 5‐FU@Zn_12_O_12_, (b) 5‐FU@CuZn_11_O_12_, (c) 5‐FU@FeZn_11_O_12_, and (d) 5‐FU@NiZn_11_O_12_ nanocomplexes formed by the adsorption of 5‐FU on Zn_12_O_12_, where the nearest atom‐to‐atom distance is indicated by the dotted line.

To further explore the interaction behavior, TM‐atoms such as Cu, Fe, and Ni were individually substituted at one Zn site in the XZn_11_O_12_ nanoclusters. Additionally, 5‐FU was adsorbed onto these TM‐doped clusters to evaluate the influence of doping on molecular interactions. The optimized structures of the TM‐doped ZnO nanoclusters (XZn_11_O_12_, where X = Zn, Cu, Fe, Ni) are shown in Figure 2a–d with the interaction distances of 2.03, 1.99, 2.00, and 2.05 Å, respectively.

#### 3.1.1 Adsorption Properties

To evaluate the stability and interaction strength of the 5‐FU molecule adsorbed onto TM‐doped ZnO nanoclusters, three key parameters were examined: total energy ($E_{\text{total}}$), adsorption energy ($E_{\mathrm{ad}}$), and dipole moment ($\mu_{D}$). The total energy provides insight into the overall stability of the system, while the adsorption energy quantifies the strength of interaction between the adsorbate and the substrate, and the dipole moment reflects changes in charge distribution and electronic polarization upon adsorption [43]. Generally, adsorption energies between −10 and −25 kcal/mol indicate strong physisorption or weak chemisorption, enabling stable yet reversible binding, whereas values more negative than −30 kcal/mol suggest chemisorption with very strong and less reversible interactions. Therefore, moderate adsorption energies are most favorable for efficient drug delivery applications.

The computed values of these parameters for pristine and TM‐doped Zn_12_O_12_ clusters are summarized in Table 1. It is evident that pristine Zn_12_O_12_ exhibits the weakest interaction with 5‐FU ($E_{\mathrm{ad}}$ = −18.56 kcal/mol), underscoring the role of TM doping in enhancing adsorption strength. Among the doped systems, the 5‐FU@FeZn_11_O_12_ complex shows the most negative adsorption energy ($E_{\mathrm{ad}}$ = −35.51 kcal/mol), indicating the strongest binding affinity. This is accompanied by a moderate dipole moment ($\mu_{D}$= 3.98 Debye), suggesting stable adsorption with moderate charge polarization. Similarly, the 5‐FU@CuZn_11_O_12_ complex exhibits substantial adsorption ($E_{\mathrm{ad}}$ = −28.69 kcal/mol), though slightly weaker than Fe, and a dipole moment of 5.11 Debye, pointing to increased electronic distortion at the interface. The 5‐FU@NiZn_11_O_12_ complex, while having a less negative adsorption energy ($E_{\mathrm{ad}}$ = −20.97 kcal/mol), presents a high dipole moment ($\mu_{D}$ = 8.08 Debye), implying pronounced charge redistribution at the interface. Thus, the increase in polarization observed after TM doping can be attributed to enhanced orbital hybridization and interfacial charge transfer, mainly involving the d orbitals of the dopants. These effects introduce electronic asymmetry at the interface and reinforce electrostatic interactions between 5‐FU and the nanocluster. As a result, larger dipole moments indicate stronger electronic coupling and improved drug–carrier interactions. Overall, Fe‐doped Zn_12_O_12_ exhibits the strongest chemisorption toward 5‐FU, ensuring high stability but potentially limiting drug release, whereas Cu‐ and Ni‐doped systems provide a favorable balance between adsorption strength, electronic response, and reversibility, making them more suitable candidates for efficient and controlled 5‐FU drug delivery.

**TABLE 1:** Total energy, adsorption energy ($E_{\mathrm{ad}}$) and dipole moment ($\mu_{D}$) of 5‐FU@TM‐doped Zn_12_O_12_ complexes.

Structure	Total energy, Hartree	$E_{\mathrm{ad}}$, kcal/mol	*μ*_D_, Debye
5‐FU	−513.984735	—	4.57
Zn_12_O_12_	−1690.689271	—	0.00
5‐FU@Zn_12_O_12_	−2204.695553	−18.56	3.54
5‐FU@CuZn_11_O_12_	−2335.230113	−28.69	5.11
5‐FU@FeZn_11_O_12_	−2262.516175	−35.51	3.97
5‐FU@NiZn_11_O_12_	−2308.367461	−20.97	8.08

### 3.2 Electronic Properties

#### 3.2.1 Frontier Molecular Orbitals (HOMO–LUMO Analysis)

Frontier molecular orbital (FMO) theory is a fundamental framework used to explain a molecule's reactivity and electronic characteristics. It focuses on two key orbitals: the highest occupied molecular orbital (HOMO), which indicates the molecule's ability to donate electrons, and the lowest unoccupied molecular orbital (LUMO), which signifies its ability to accept electrons [44]. A key parameter in FMO theory is the HOMO–LUMO energy gap ($E_{g}$), which quantifies the energy required to excite an electron from the HOMO to the LUMO. It is calculated using the relation: $E_{g}=E_{\text{LUMO}}-E_{\text{HOMO}}$, where $E_{\text{HOMO}}$ and $E_{\text{LUMO}}$ denote the orbital energies of the HOMO and LUMO, respectively. Generally, HOMO and LUMO orbitals are visually represented using two colors: green for the positive phase and red for the negative phase. A smaller HOMO–LUMO gap typically indicates increased chemical reactivity, greater polarizability, and decreased kinetic stability [45].

**FIGURE 3:**
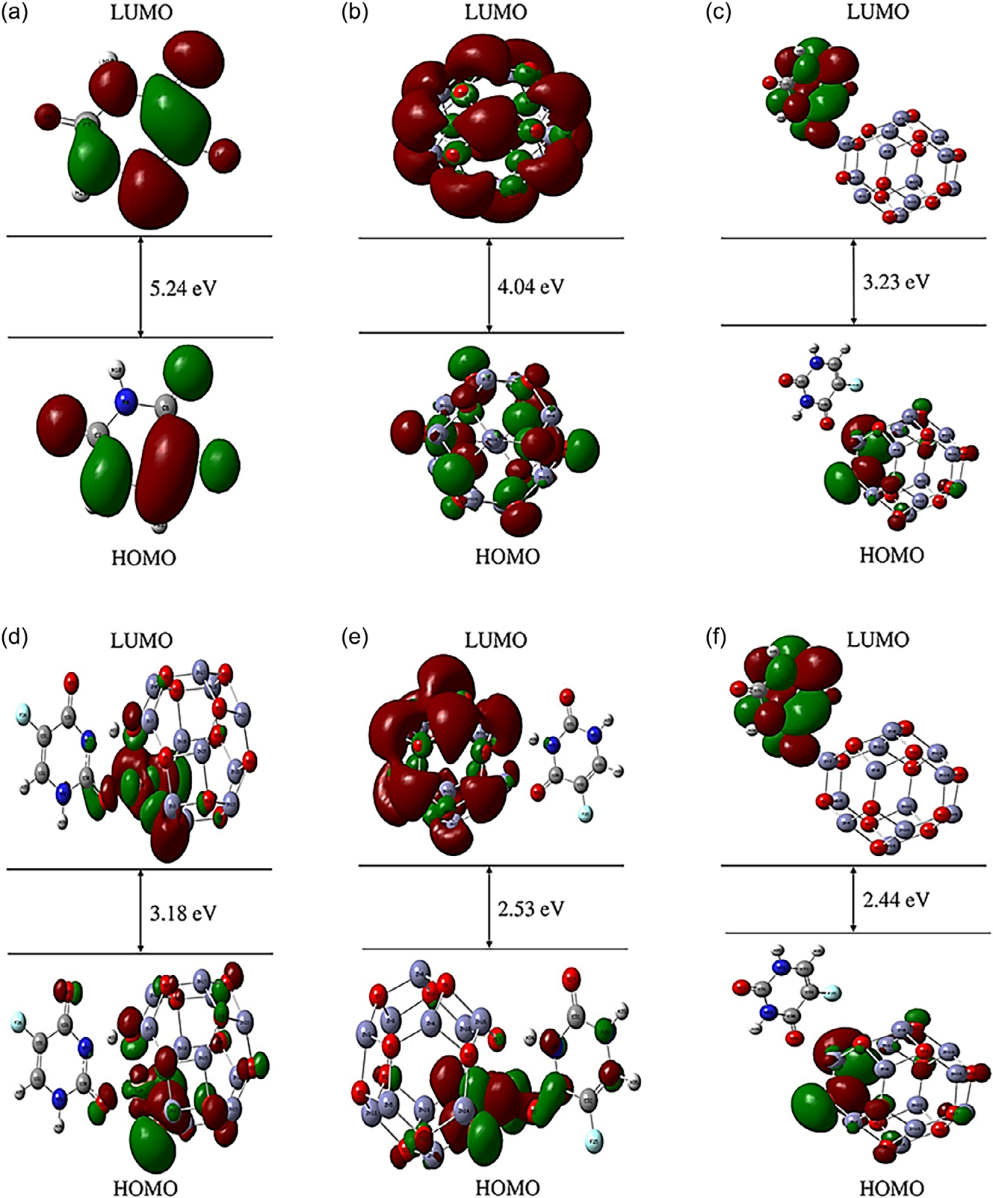
HOMO–LUMO plots with energy gaps for (a) 5‐FU, (b) pristine Zn_12_O_12_, (c) 5‐FU@Zn_12_O_12_, (d) 5‐FU@CuZn_11_O_12_, (e) 5‐FU@FeZn_11_O_12_, and (f) 5‐FU@NiZn_11_O_12_ nanocomplexes.

The FMOs of 5‐FU, pristine Zn_12_O_12_, and their doped complexes are illustrated in Figure 3, highlighting notable changes in electronic behavior upon adsorption and doping in the gaseous phase. The isolated 5‐FU molecule (Figure 3a) shows a wide HOMO–LUMO gap of 5.24 eV, indicating high electronic stability and low reactivity. In pristine Zn_12_O_12_ (Figure 3b), a narrower gap of 4.04 eV is observed, with both HOMO and LUMO symmetrically delocalized across the Zn–O framework, suggesting moderate reactivity. Adsorption of 5‐FU onto ZnO (Figure 3c) further reduces the gap to 3.23 eV. Notably, doping with TMs significantly alters the electronic structure: the Cu‐doped complex (5‐FU@CuZn_11_O_12_) exhibits a gap of 3.18 eV (Figure 3d); Fe doping (5‐FU@FeZn_11_O_12_) lowers it to 2.53 eV (Figure 3e); and the Ni‐doped system (5‐FU@NiZn_11_O_12_) shows the smallest gap of 2.44 eV (Figure 3f), reflecting the highest electronic reactivity among all configurations. This pronounced gap reduction arises from the introduction of localized d‐orbital states by TM dopants, which enhance orbital mixing and facilitate interfacial charge transfer between the nanocluster and 5‐FU, thereby strengthening drug–carrier interactions. Consequently, the reduced HOMO–LUMO gap not only reflects increased chemical reactivity but also supports the improved binding affinity of the doped ZnO nanoclusters. The calculated gaps are in close agreement with previously reported values for 5‐FU, Zn_12_O_12_, 5‐FU@Zn_12_O_12_, and 5‐FU@FeZn_11_O_12_ [12].

To validate the B3LYP results, we performed additional calculations on 5‐FU@NiZn_11_O_12_ using the higher‐level WB97XD functional with the LANL2DZ basis set and compared the adsorption energy and HOMO–LUMO gap. WB97XD yielded *E*_ad_ = −24.59 kcal/mol and a HOMO–LUMO gap of 6.65 eV, while B3LYP gave −20.97 kcal/mol and 2.44 eV, respectively. Despite the slightly larger gap predicted by WB97XD due to dispersion corrections, the overall structural features, interaction trends, and relative strengths were consistent with B3LYP results, confirming the reliability of our chosen functional and basis set.

#### 3.2.2 Density of States

**FIGURE 4:**
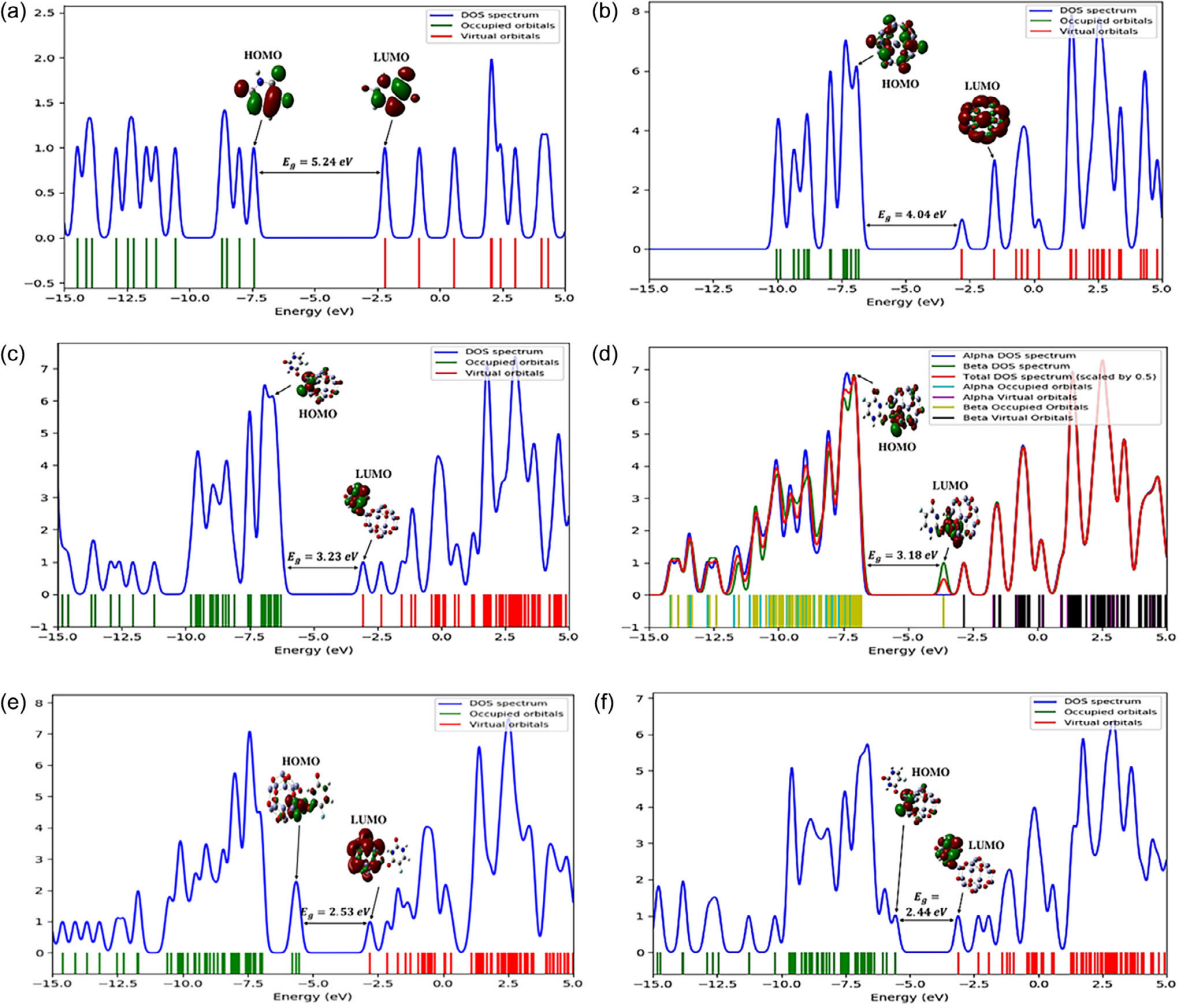
DOS spectra for (a) 5‐FU, (b) pristine Zn_12_O_12_, (c) 5‐FU@Zn_12_O_12_, (d) 5‐FU@CuZn_11_O_12_, (e) 5‐FU@FeZn_11_O_12_, and (f) 5‐FU@NiZn_11_O_12_, computed using the B3LYP/LANL2DZ method.

The DOS spectrum provides a comprehensive distribution of electronic states across various energy levels, revealing how many states are available for occupation at each level. While FMO analysis focuses on the discrete energies of the frontier orbitals, DOS offers a more continuous and detailed view, particularly valuable for systems with densely packed or nearly degenerate orbitals. The computed DOS spectra of 5‐FU, pristine Zn_12_O_12_, and their doped complexes are presented in Figure 4, where occupied orbitals are shown in green (indicating filled states) and virtual (unoccupied) orbitals in red (representing states available for excitation). Upon adsorption and doping, significant redistribution of electronic states is observed near the Fermi level, along with the emergence of new mid‐gap states, indicating pronounced orbital hybridization between 5‐FU and the ZnO framework. These features reveal enhanced electronic coupling and charge–transfer interactions, which rationalize the reduced band gaps and increased reactivity of the complexes. Notably, the energy gaps estimated from DOS closely align with those from FMO analysis, reinforcing the consistency, and reliability of the electronic structure findings.

#### 3.2.3 Global Reactivity Descriptors

Global reactivity descriptors provide critical insight into the electronic structure and inherent properties of molecular systems, playing a key role in the evaluation and prediction of their chemical reactivity [46]. These descriptors, namely the HOMO–LUMO energy gap ($E_{g}$), electronegativity (χ), chemical potential (μ), chemical hardness (η), softness (S), and electrophilicity index (ω), are calculated using the energies of the FMOs ($E_{\text{HOMO}}$ and $E_{\text{LUMO}}$). The mathematical expressions used to compute these descriptors are presented in Equations (2–, 3) [46, 47], and the computed values are summarized in Table 2.

(2) $$E_{g}=E_{\text{LUMO}}-E_{\text{HOMO}},\;\;\chi =-\frac{1}{2}\left(E_{\text{HOMO}}+E_{\text{LUMO}}\right),\;\;\mu =\frac{1}{2}(E_{\text{HOMO}}+E_{\text{LUMO}})$$

(3) $$\eta =\frac{1}{2}\left(E_{\text{LUMO}}-E_{\text{HOMO}}\right),\;\;\;S=\frac{1}{2\eta },\;\;\;\omega =\frac{\mu^{2}}{2\eta }$$

**TABLE 2:** Calculated $E_{\text{LUMO}}$, $E_{\text{HOMO}}$, energy gap ($E_{g}$), electronegativity (χ), chemical potential (μ), hardness (η), softness (S), and electrophilicity index (ω) for all systems. All parameters are in eV, whereas S is in (eV)^−1^.

System	$E_{\text{LUMO}}$	$E_{\text{HOMO}}$	$E_{g}$	*χ*	*μ*	*η*	S	*ω*
5‐FU	−2.18	−7.42	5.24	4.80	−4.80	2.62	0.19	4.39
Zn_12_O_12_	−2.79	−6.83	4.04	4.81	−4.81	2.01	0.24	5.74
5‐FU@Zn_12_O_12_	−3.04	−6.27	3.23	4.66	−4.66	1.61	0.31	6.73
5‐FU@CuZn_11_O_12_	−3.61	−6.79	3.18	5.20	−5.20	1.59	0.32	8.51
5‐FU@FeZn_11_O_12_	−2.80	−5.33	2.53	3.83	−3.83	1.21	0.40	6.01
5‐FU@NiZn_11_O_12_	−3.11	−5.55	2.44	4.33	−4.33	1.22	0.41	7.68

Molecules possessing a higher HOMO–LUMO energy gap are typically characterized by greater chemical hardness, enhanced stability, and reduced reactivity. In contrast, species with a smaller energy gap exhibit increased softness and higher reactivity [48]. This reciprocal relationship between η and S is clearly observed in 5‐FU and its ZnO‐doped complexes. The pristine 5‐FU molecule, with a larger $E_{g}$ (5.24 eV), exhibits a higher η value (2.62 eV) and lower S value (0.19 eV^−1^), indicating greater chemical stability and reduced reactivity. In contrast, the doped complexes 5‐FU@FeZn_11_O_12_ and 5‐FU@NiZn_11_O_12_ display narrower band gaps of 2.53 and 2.44 eV, respectively, along with lower η values (1.21 and 1.22 eV) and higher S values (0.40 eV^−1^ and 0.41 eV^−1^). These results confirm that a reduced energy gap leads to decreased η and increased S, making the doped systems more chemically reactive and less stable.

Moreover, the adsorption of 5‐FU onto Zn_12_O_12_‐based nanocages leads to an increase in the electrophilicity index for all resulting complexes, indicating an enhanced electrophilic character [4]. Generally, a higher ω value suggests a stronger tendency to act as an electrophile, while a lower ω value is typically associated with better nucleophilic behavior [49]. Among the studied systems, 5‐FU@CuZn_11_O_12_ (ω = 8.51 eV) and 5‐FU@NiZn_11_O_12_ (ω = 7.68 eV) exhibited the highest electrophilicity index, making them the strongest electrophiles compared to the pristine Zn_12_O_12_ (ω = 5.74 eV) and 5‐FU (ω = 4.39 eV), which displayed nucleophilic character. This trend is further supported by the electronic chemical potential, which reflects the escaping tendency of electrons from a system. Generally, lower (more negative) values of μ are associated with stronger electrophilic nature, as the system is more inclined to accept electrons. In this context, 5‐FU@FeZn_11_O_12_ and 5‐FU@NiZn_11_O_12_, with μ values of –3.83 and –4.33 eV, respectively, demonstrated higher electrophilic tendencies compared to pristine Zn_12_O_12_ (–4.81 eV) and 5‐FU (–4.80 eV). These results underscore the significant role of TM doping in enhancing the electrophilic nature of Zn_12_O_12_ nanocages compared to pristine Zn_12_O_12_ and isolated 5‐FU. Overall, the reduced hardness, increased softness, and enhanced electrophilicity of the TM‐doped Zn_12_O_12_ nanoclusters, compared to pristine Zn_12_O_12_ and isolated 5‐FU, indicate stronger charge–transfer capability, leading to improved adsorption and interfacial interactions, thereby making them highly favorable for efficient drug loading, transport, and controlled release.

#### 3.2.4 UV‐Visible Spectral Analysis

**FIGURE 5:**
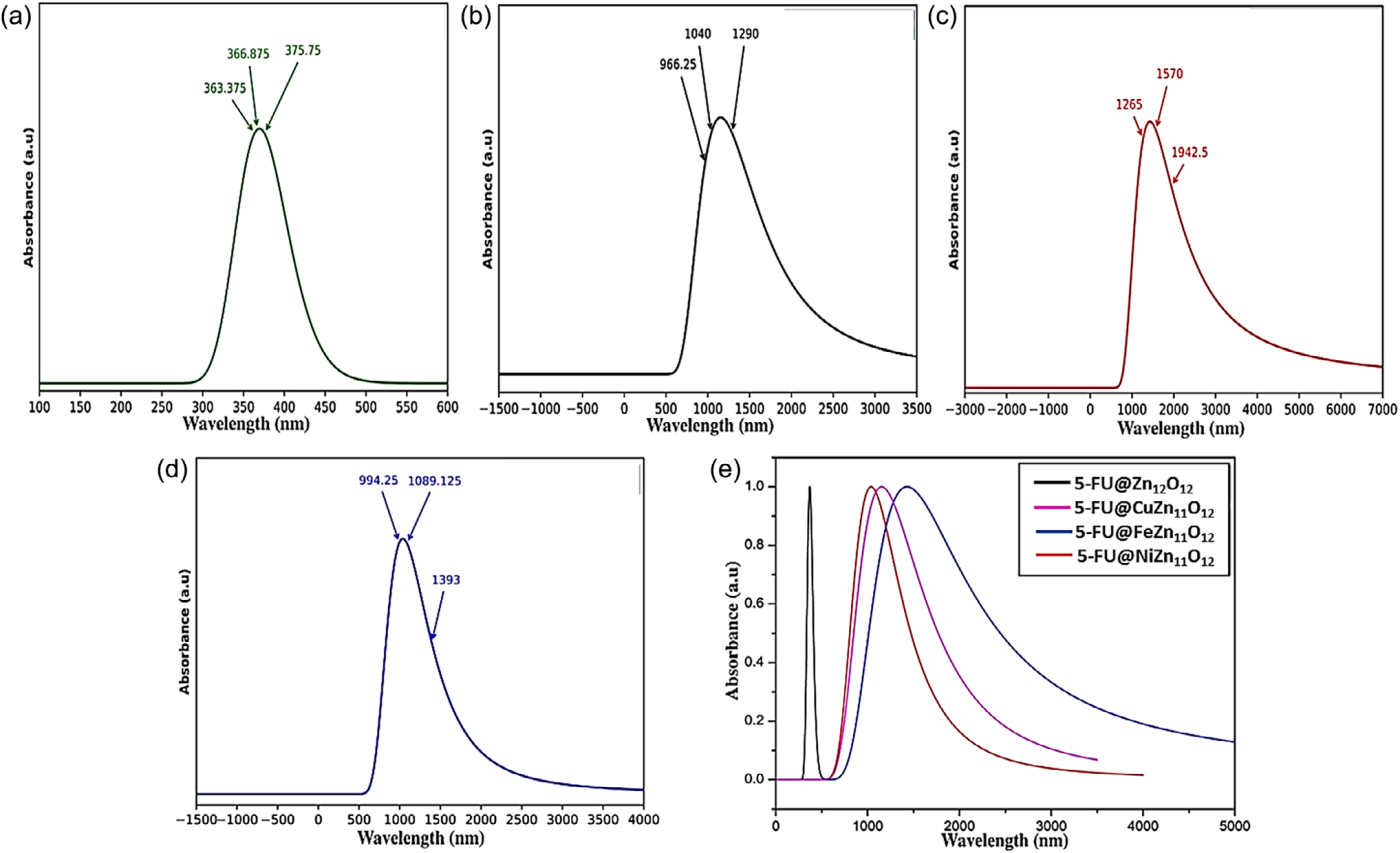
UV–visible absorption spectra of (a) 5‐FU@Zn_12_O_12_, (b) 5‐FU@CuZn_11_O_12_, (c) 5‐FU@FeZn_11_O_12_, (d) 5‐FU@NiZn_11_O_12_, and (e) combined spectra of these complexes.

UV–visible spectra were analyzed to investigate the electronic absorption behavior of pristine and TM‐doped ZnO nanoclusters upon 5‐FU adsorption. The absorption wavelengths ($\lambda_{\max}$), oscillator strengths (f), and the major contributing orbitals of 5‐FU@Zn_12_O_12_ and 5‐FU@TM‐doped ZnO are summarized in Table 3. The corresponding spectra are presented in Figure 5.

**TABLE 3:** Calculated wavelengths of maximum absorption ($\lambda_{\max}$) with major contribution of orbitals and oscillator strengths of different complexes.

System	Calculated *λ*_max_, nm	Oscillator strength (f)	Orbital description for major contributions
5‐FU@Zn_12_O_12_	375.75	0.0035	HOMO → LUMO (96%)
	366.84	0.0024	H‐1 → LUMO (96%)
	363.39	0.0025	H‐2 → LUMO (97%)
5‐FU@CuZn_11_O_12_	1289.88	0.0022	HOMO(B) → LUMO(B) (26%),
			H‐7(B) → LUMO(B) (17%)
	1040.57	0.0007	H‐8(B) → LUMO(B) (24%),
			HOMO(B) → LUMO(B) (15%)
	966.13	0.0010	HOMO(B) → LUMO(B) (17%),
			H‐8(B) → LUMO(B) (13%)
5‐FU@FeZn_11_O_12_	1941.75	0.0001	H‐1 → L + 5 (53%),
			H‐1 → L + 2 (22%)
	1569.65	0.0006	HOMO → L + 5 (38%),
			HOMO → L + 2 (16%)
	1266.58	0.0006	H‐2 → L + 5 (33%),
			H‐2 → L + 2 (16%)
5‐FU@NiZn_11_O_12_	1392.92	0.0004	H‐2 → L + 5 (33%),
			H‐2 → L + 2 (16%)
	1088.91	0.0020	H‐1 → LUMO (31%),
			H‐1 → L + 2 (40%)
	994.49	0.0035	H‐2 → LUMO (27%),
			H‐2 → L + 2 (38%)

For the 5‐FU@Zn_12_O_12_ complex, three maximum absorption wavelengths were identified in the near‐UV region at 375.75, 366.84, and 363.39 nm, with corresponding oscillator of 0.0035, 0.0024, and 0.0025, respectively. These transitions originate primarily from excitations involving the HOMO, HOMO−1, and HOMO−2 to the LUMO, indicating strong *π*–π* and efficient charge transfer between 5‐FU and the pristine Zn_12_O_12_nanocage. In the 5‐FU@CuZn_11_O_12_ complex, the absorption maxima were red‐shifted into the near‐infrared (NIR) region at 1289.88, 1040.57, and 966.13 nm, with oscillator strengths of 0.0022, 0.0007, and 0.0010, respectively. These transitions involve excitations from deeper occupied orbitals (H−7(B), H−8(B), HOMO(B)) to the LUMO(B), indicating significant charge redistribution and a modified electronic environment upon Cu doping.

5‐FU@FeZn_11_O_12_, even longer maximum wavelengths were observed at 1941.75, 1569.65, and 1266.58 nm, with very low oscillator strengths (0.0001, 0.0006, and 0.0006), placing these transitions deep in the infrared (IR) region. These transitions involved excitations from H−1, HOMO, and H−2 to higher unoccupied orbitals such as L + 5 and L + 2. The pronounced red shift and weak intensities suggest the presence of *d*–*d* transitions and enhanced electron delocalization due to Fe doping. In contrast, the 5‐FU@NiZn_11_O_12_ complex exhibits absorption maxima at 1392.92, 1088.91, and 994.49 nm, with moderate oscillator strengths of 0.0004, 0.0020, and 0.0035, respectively, in the NIR region. These transitions arise from multiple orbital excitations involving HOMO, H−1, and H−2 to LUMO and L + 2, reflecting significant charge transfer facilitated by Ni incorporation.

Overall, the near‐UV absorption in the pristine system primarily arises from *π* → π* transitions of 5‐FU and HOMO → LUMO excitations within the ZnO framework, whereas TM doping introduces metal‐centered d states near the Fermi level, leading to low‐energy charge–transfer transitions that are responsible for the pronounced red shift into the visible and near‐infrared regions. The observed red shifts (i.e., 1289.88 nm for Cu‐doped, 1941.75 nm for Fe‐doped, and 1392.92 nm for Ni‐doped complexes) quantitatively correlate with the corresponding reduction in HOMO–LUMO gaps (3.23 eV → 3.18, 2.53, and 2.44 eV, respectively), confirming that dopant‐induced electronic structure changes are responsible for the low‐energy optical transitions and enhanced charge–transfer interactions in the doped systems. Among the studied systems, 5‐FU@NiZn_11_O_12_ stands out as the most promising candidate due to its balanced characteristics, i.e., significant redshift, strong oscillator strength, and well‐defined HOMO–LUMO transitions, suggesting enhanced electronic interactions, optical activity, and favorable transition probabilities upon 5‐FU adsorption.

#### 3.2.5 Molecular Electrostatic Potential

MEP visualizes the charge distribution within a molecule to identify nucleophilic and electrophilic sites, evaluate reactivity, and analyze intermolecular interactions. In a molecular system with electron density $\rho (\vec{r})$, the electrostatic potential $V^{\mathrm{ES}}(\vec{r})$ at a given point $\vec{r}$ is determined as $V^{\mathrm{ES}}\left(\vec{r}\right)=\sum_{A}\frac{Z_{A}}{\left|{\vec{R}}_{A}-\vec{r}\right|}-\int \frac{\rho ({\vec{r}}^{\prime })}{\left|\vec{r}\prime -\vec{r}\right|}d{\vec{r}}^{\prime }$, where $Z_{A}$ denotes the charge of nucleus A located at position ${\vec{R}}_{A}$, and the first term on the right corresponds to the nuclear contribution, while the second term represents the electronic contribution [50]. In the MEP map, the electrostatic potential increases in the order: red < orange < yellow < green < blue, where red regions indicate high electron density (negative potential) favorable for electrophilic attack, while blue regions show low electron density (positive potential) favorable for nucleophilic attack, and yellow/green regions represent areas of neutral potential [51].

**FIGURE 6:**
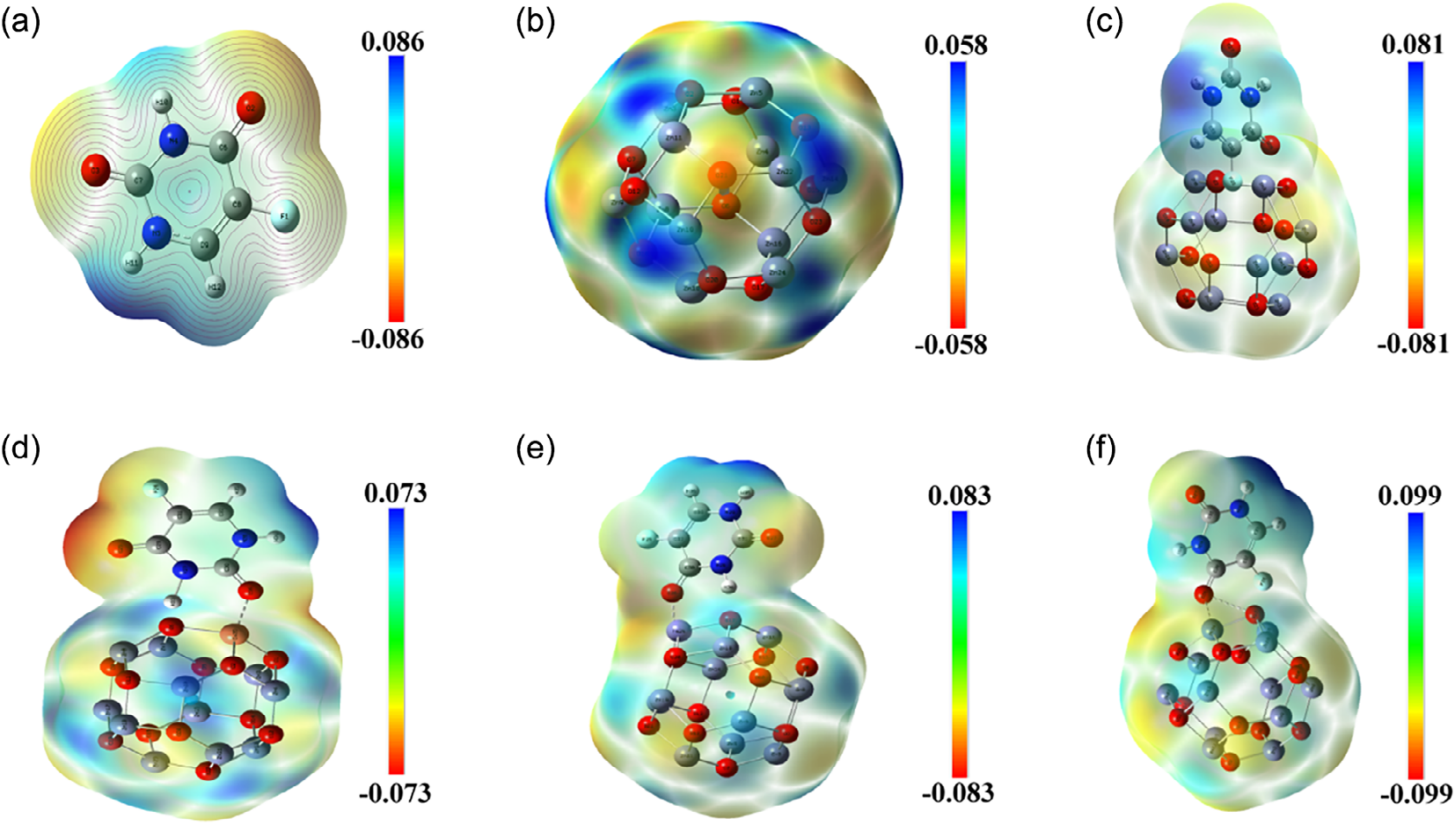
MEP surfaces of (a) 5‐FU, (b) pristine Zn_12_O_12_, (c) 5‐FU@Zn_12_O_12_, (d) 5‐FU@CuZn_11_O_12_, (e) 5‐FU@FeZn_11_O_12_, and (f) 5‐FU@NiZn_11_O_12_.

As evident from Figure 6, the 5‐FU@NiZn_11_O_12_ complex exhibited a broader electrostatic potential distribution range (from –0.099 to 0.099 a.u.) compared to other TM‐doped complexes, stronger charge polarization upon adsorption. In this complex, the maximum positive potential regions were predominantly located on hydrogen atoms (H34 and H35), marking these as potential electrophilic attack sites. Conversely, the negative potential was mainly concentrated on oxygen atoms (O2 and O7), indicating likely nucleophilic sites due to higher electron density. A similar trend was observed in the pristine 5‐FU@Zn_12_O_12_ complex, where the highest positive potential was localized on H34 and H35, while negative regions were found on O1 and O2. In the case of 5FU@CuZn_11_O_12_, H34 and H35 served as electrophilic centers, while O15, O21, and O23 showed nucleophilic behavior. For the 5‐FU@FeZn_11_O_12_ complex, the maximum positive potential appeared on H34 and H35, whereas O1, O2, and O7 were identified as potential nucleophilic sites. Overall, TM doping clearly enhances charge polarization compared to pristine Zn_12_O_12_, as reflected by the stronger separation of positive and negative regions. In particular, the Ni‐ and Fe‐doped systems show the highest polarization, indicating stronger electrostatic interactions and higher reactivity toward 5‐FU, which supports their improved adsorption and dipole moment behavior.

#### 3.2.6 Mulliken Atomic Charges

**FIGURE 7:**
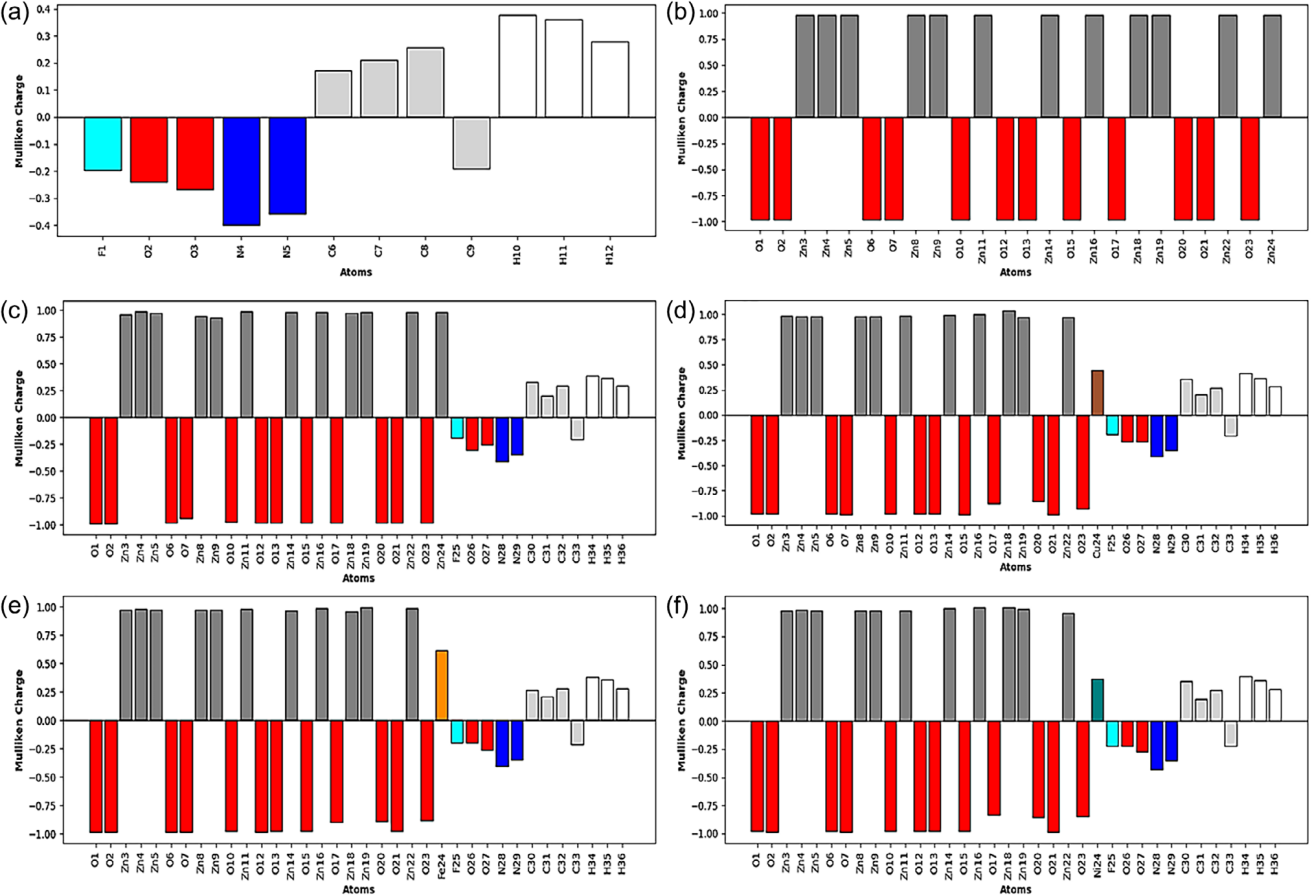
Mulliken atomic charges of (a) 5‐FU, (b) pristine Zn_12_O_12_, (c) 5‐FU@Zn_12_O_12_, (d) 5‐FU@CuZn_11_O_12_, (e) 5‐FU@FeZn_11_O_12_, and (f) 5‐FU@NiZn_11_O_12_.

Mulliken atomic charge analysis provides insights into electron distribution within molecules and aids in evaluating key properties such as dipole moment, polarizability, and electronic structure [29]. It reveals that oxygen atoms bear negative charges, hydrogen atoms carry positive charges, and carbon atoms exhibit either positive or negative charges depending on their positions within the molecular framework. The Mulliken charge values of 5‐FU and the nanocomplexes are shown in Figure 7.

In the 5‐FU@NiZn_11_O_12_ complex, atoms such as Zn3, Zn4, Zn5, Zn9, Zn11, Zn14, Zn16, Zn18, Zn19, and the drug‐related atoms C30, H34, and H35 exhibited more positive charges, indicating enhanced electrophilic character. In contrast, atoms like O1, O2, O6, O7, O12, O15, and O21 carried negative charges, suggesting nucleophilic behavior. Similarly, in the 5‐FU@FeZn_11_O_12_ complex, electrophilic sites include Zn3, Zn4, Zn9, Zn11, Zn16, Zn19, C32, H34, and H35, while O1, O2, O6, O7, O10, O12, and O15 acted as nucleophilic centers. For the 5‐FU@CuZn_11_O_12_ complex, more positive charges were observed on Zn3, Zn4, Zn5, Zn8, Zn9, Zn11, Zn14, Zn16, Zn18, C30, H34, and H35, whereas nucleophilic regions comprised O5, O9, O11, O12, O14, O15, O20, O21, and O23. In the 5‐FU@Zn_12_O_12_ complex, electrophilic atoms were identified as Zn4, Zn11, Zn14, Zn22, C30, H34, and H35, whereas O1, O2, O12, O13, O15, O17, O20, and O23 displayed nucleophilic tendencies. These charge distributions likely facilitated hydrogen bond formation, particularly where highly positive hydrogen atoms and highly negative oxygen atoms were in close spatial proximity. Although Mulliken atomic charges are known to be sensitive to the choice of basis set, all systems in this study were computed at the same theoretical level. Therefore, the relative variations in charge distribution remain meaningful and reliably reflect the influence of TM doping. Moreover, these qualitative trends are consistent with the MEP maps and dipole moment analysis, supporting the observed enhancement of interfacial interactions.

#### 3.2.7 QTAIM Analysis

The QTAIM, introduced by Bader et al. [52] provides a powerful framework to investigate intra‐ and intermolecular interactions through the analysis of bond critical points (BCPs). In the present study, QTAIM was applied to explore the nature of bonding between the drug 5‐FU and the XZn_11_O_12_ complexes by evaluating the topological parameters at the critical points. The parameters include the electron density ρ(r), its Laplacian $\nabla^{2}\rho (r)$, potential energy density V(r), kinetic energy density G(r), total energy density H(r), and the energy ratio G(r)/|V(r)| as depicted in Table 4. These parameters are well established within QTAIM theory, where a negative Laplacian ($\nabla^{2}\rho (r)$ < 0) and negative total energy density (H(r) < 0) indicate shared‐shell (covalent) interactions, whereas positive $\nabla^{2}\rho (r)$ and H(r) values are characteristic of closed‐shell, electrostatic interactions. Furthermore, the ratio G(r)/|V(r)| serves as a diagnostic tool, with G(r)/|V(r)|< 0.5 denoting covalent bonds, 0.5–1.0 suggesting partially covalent interactions, and values greater than 1.0 representing closed‐shell, NCI [53].

**TABLE 4:** Calculated topological parameters: electron density *ρ*(*r*), its Laplacian $\nabla^{2}\rho (r)$, potential energy density V(r), kinetic energy density G(r), total energy density H(r), and the ratio G(r)/|V(r)|for the complexes at BCPs in a.u.

Complexes	BCPs	*ρ* (r)	$\nabla^{2}$ *ρ*(*r*)	V(r)	G(r)	H(r)	G(r)/|V(r)|
5‐FU@ Zn_12_O_12_	Zn24‐O26	0.0659	0.3816	−0.0953	0.0850	−0.0102	0.8927
	O20‐H34	0.2258	−0.7716	−0.3500	0.0785	−0.2715	0.2244
5‐FU@CuZn_11_O_12_	Cu24‐O26	0.0681	0.5020	−0.1212	0.1234	0.0021	1.0182
	O20‐H34	0.2283	−0.7877	−0.3541	0.0786	−0.2755	0.2220
5‐FU@FeZn_11_O_12_	Fe24‐O26	0.0645	0.4504	−0.1225	0.1175	−0.0050	0.9592
	O17‐H34	0.0834	0.1806	−0.0922	0.0235	−0.0235	0.2547
5‐FU@NiZn_11_O_12_	Ni24‐O26	0.0537	0.4017	−0.0902	0.0953	0.0051	1.0565
	Zn16‐F25	0.0221	0.0995	−0.0266	0.0017	−0.0017	0.0631

Table 4 provides topological insight into the bonding characteristics of 5‐FU adsorption on TM‐doped Zn_12_O_12_ complexes. For Zn‐ and Cu‐doped systems, the hydrogen‐bond BCPs (O23–F25 and O20–H34) exhibit relatively high electron densities [ρ(r) ≈ 0.23 a.u.], negative $\nabla^{2}\rho (r)$ and H(r) values, and low G(r)/|V(r)| ratios (∼0.22), confirming strong covalent contributions. In comparison, the Fe‐ and Ni‐doped systems display lower ρ(r) values with positive $\nabla^{2}\rho (r)$, suggesting weaker covalent interactions. In contrast, the metal–oxygen BCPs across all complexes show consistently low ρ(r) values (0.0659, 0.0681, 0.0645, and 0.0537 a.u. for 5‐FU@Zn_12_O_12_and Cu‐, Fe‐, and Ni‐doped Zn_12_O_12_, respectively) and G(r)/|V(r)| ratios close to or exceeding unity (0.8929, 1.0182, 0.9592, and 1.0565, respectively), indicating closed–shell, electrostatic interactions. Overall, these results highlight that Zn‐ and Cu‐doped complexes are dominated by stronger covalent hydrogen‐bond interactions, as indicated by higher ρ(r) and negative H(r) at the BCPs, whereas Fe‐ and Ni‐doped systems exhibit predominantly closed‐shell, electrostatic interactions characterized by lower *ρ* (*r*) and G/|V| ratios near or above unity. This transition from covalent‐dominated to mixed electrostatic–covalent bonding explains the observed differences in adsorption strength and stability among the doped Zn_12_O_12_ nanoclusters.

#### 3.2.8 NCI/RDG Analysis

**FIGURE 8:**
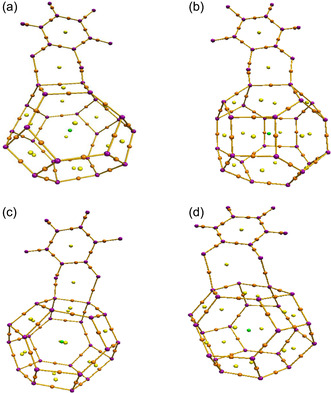
QTAIM molecular graphs of (a) 5‐FU@Zn_12_O_12_, (b) 5‐FU@CuZn_11_O_12_, (c) 5‐FU@FeZn_11_O_12_, and (d) 5‐FU@NiZn_11_O_12_, highlighting the BCPs.

**FIGURE 9:**
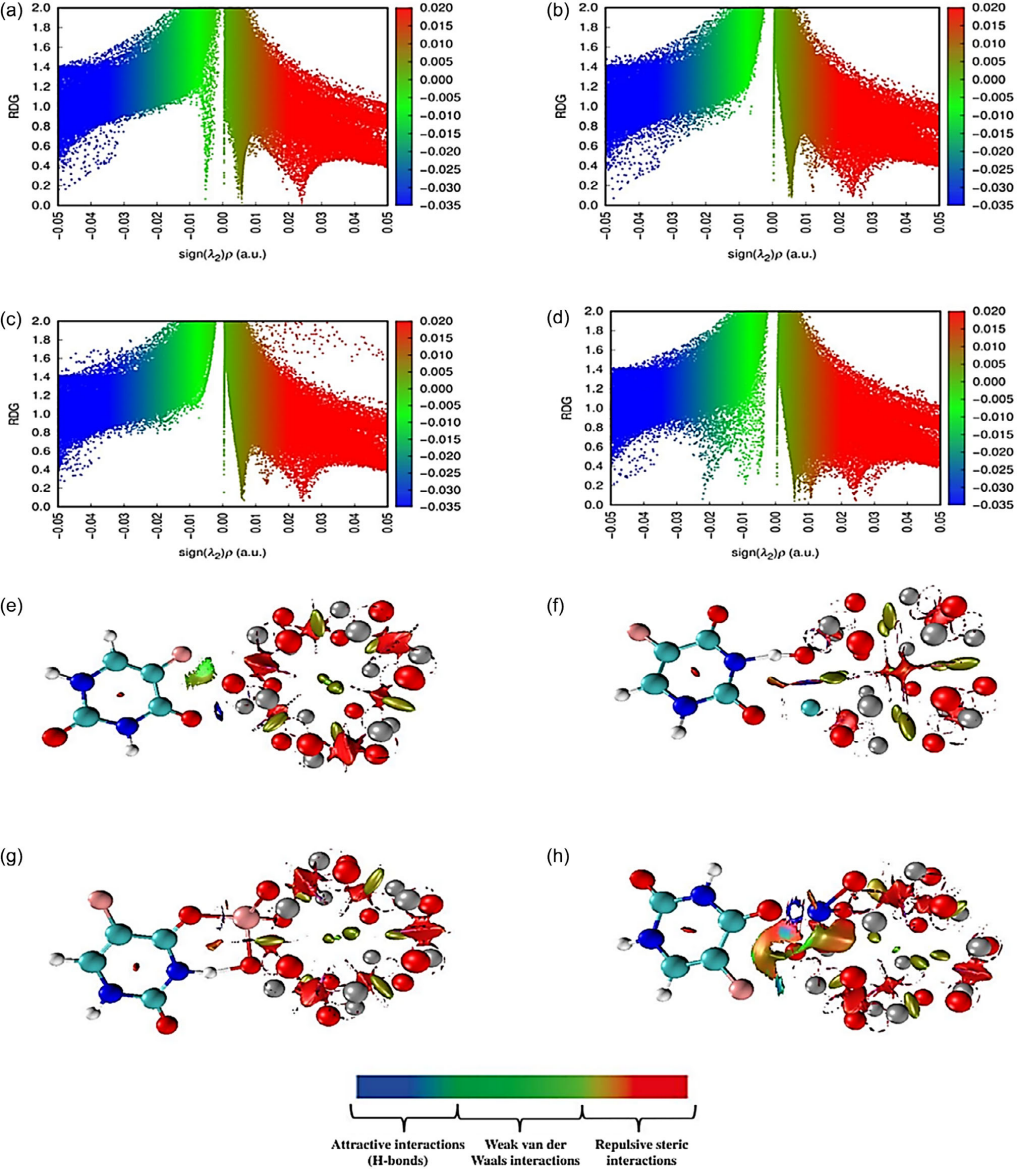
(a–d) RDG versus sign(*λ*_2_)*ρ* plots, and (e–h) color‐filled NCI isosurfaces for 5‐FU@Zn_12_O_12_, 5‐FU@CuZn_11_O_12_, 5‐FU@FeZn_11_O_12_, and 5‐FU@NiZn_11_O_12_, respectively.

NCI analysis, an extension of the QTAIM framework, employs the reduced density gradient (RDG) to effectively visualize and distinguish different types of NCI [54]. RDG is a dimensionless quantity, defined as: $s=\frac{1}{2{\left(3\pi^{2}\right)}^{1/3}}\frac{\left|\nabla \rho \right|}{\rho^{4/3}}$ where ρ is the electron density and ∇ρ corresponds to its first derivative [55]. RDG analysis also relies on the product sign(*λ*_2_)*ρ*, where *λ*_2_ is the second eigenvalue of the electron density Hessian matrix [56]. The NCI/RDG plots of the studied nanocomplexes, shown in Figure 8, allow identification of different interaction types: attractive interactions (such as hydrogen bonds) correspond to sign(*λ*_2_)*ρ* < 0 and are depicted in blue; weak van der Waals (vdW) interactions correspond to sign(*λ*_2_)*ρ* ≈ 0 and appear in green; while repulsive steric interactions correspond to sign(*λ*_2_)*ρ* > 0 and are shown in red [53, 56]. Figure 9 clearly shows the interaction patterns in the studied complexes, while the corresponding NCI isosurface plots below further illustrate these interactions in 3D space. In 5‐FU@Zn_12_O_12_ (Figure 9a), blue scattered points in the negative region indicate strong hydrogen bonding, complemented by spikes between –0.01 and 0.01 a.u. representing weak vdW forces, and positive spikes at 0.02–0.03 a.u. showing low steric repulsion. For 5‐FU@CuZn_11_O_12_ (Figure 9b), fewer vdW peaks and red spikes between 0.01 and 0.03 a.u. highlight enhanced steric effects. Similarly, 5‐FU@FeZn_11_O_12_ (Figure 9c) exhibits limited negative scattered points and vdW contributions, with prominent positive spikes indicating weaker hydrogen bonding and dominant repulsion. In contrast, 5‐FU@NiZn_11_O_12_ (Figure 9d) displays negative scattered points with features between –0.03 and –0.02 a.u., additional vdW contributions up to 0.00 a.u., and steric peaks at 0.01–0.03 a.u., reflecting a well‐balanced combination of attractive and repulsive forces. The NCI isosurfaces also visually confirm these trends, explaining the superior adsorption observed for Ni‐doped Zn_12_O_12_, consistent with the adsorption results.

##### 3.2.8.1 Molecular Docking

Based on the DFT results, 5‐FU@NiZn_11_O_12_ was selected for molecular docking because it exhibits the most favorable balance between adsorption strength, the highest dipole moment, and a reduced HOMO–LUMO gap, ensuring stable yet reversible drug–carrier interactions. This makes it the most suitable and relevant candidate for evaluating protein binding behavior with HSA. In molecular docking, binding energy reflects the strength of interaction between the nanocomplex and HSA, where a lower value indicates stronger and more stable molecular bonds [57].

Without altering the shape of the Zn_12_O_12_ nanocage by fixing the rotatable bonds with torsion, the results from molecular docking indicated that 5‐FU@NiZn_11_O_12_ nanocomplex binds to HSA with a lower binding energy of –5.36 kcal/mol, compared to –3.62 kcal/mol for 5‐FU. This slightly increased affinity was also reflected in the calculated inhibition constant ($K_{i}$), where $K_{i}$ dropped from 2.23 mM (for 5‐FU) to 117.15 μM (for 5‐FU@NiZn_11_O_12_), suggesting a stable and stronger interaction upon nanocomplexation. From a pharmacological perspective, binding energies in the range of −5 to −7 kcal/mol correspond to moderate yet biologically relevant protein–ligand interactions. The enhanced binding of 5‐FU@NiZn_11_O_12_ relative to free 5‐FU indicates improved molecular recognition upon nanocomplexation. Such moderate affinity is favorable for HSA‐mediated transport, as it supports stable circulation while allowing reversible drug release, highlighting the suitability of 5‐FU@NiZn_11_O_12_ as a drug‐delivery candidate.

**FIGURE 10:**
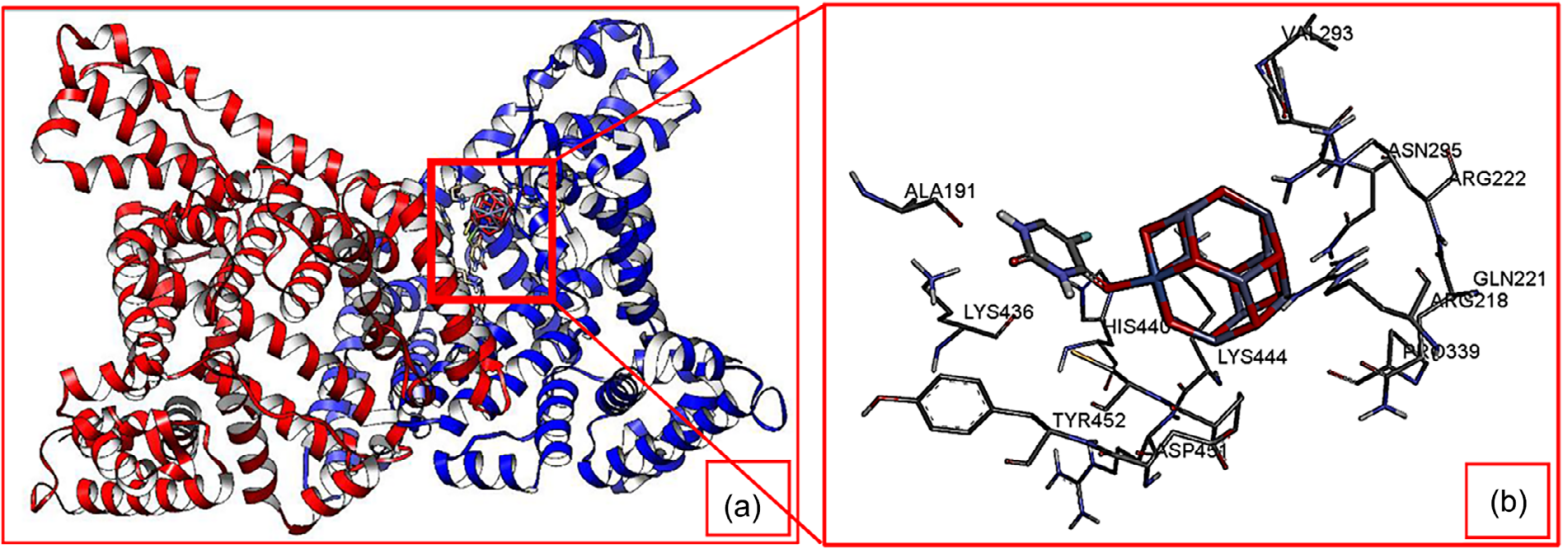
(a) Flat ribbon view of the HSA protein bound with the 5‐FU@NiZn_11_O_12_ nanocomplex, and (b) active amino acid residues in the ligand‐binding domain of the HSA protein interacting with the 5‐FU@NiZn_11_O_12_ nanocomplex.

**FIGURE 11:**
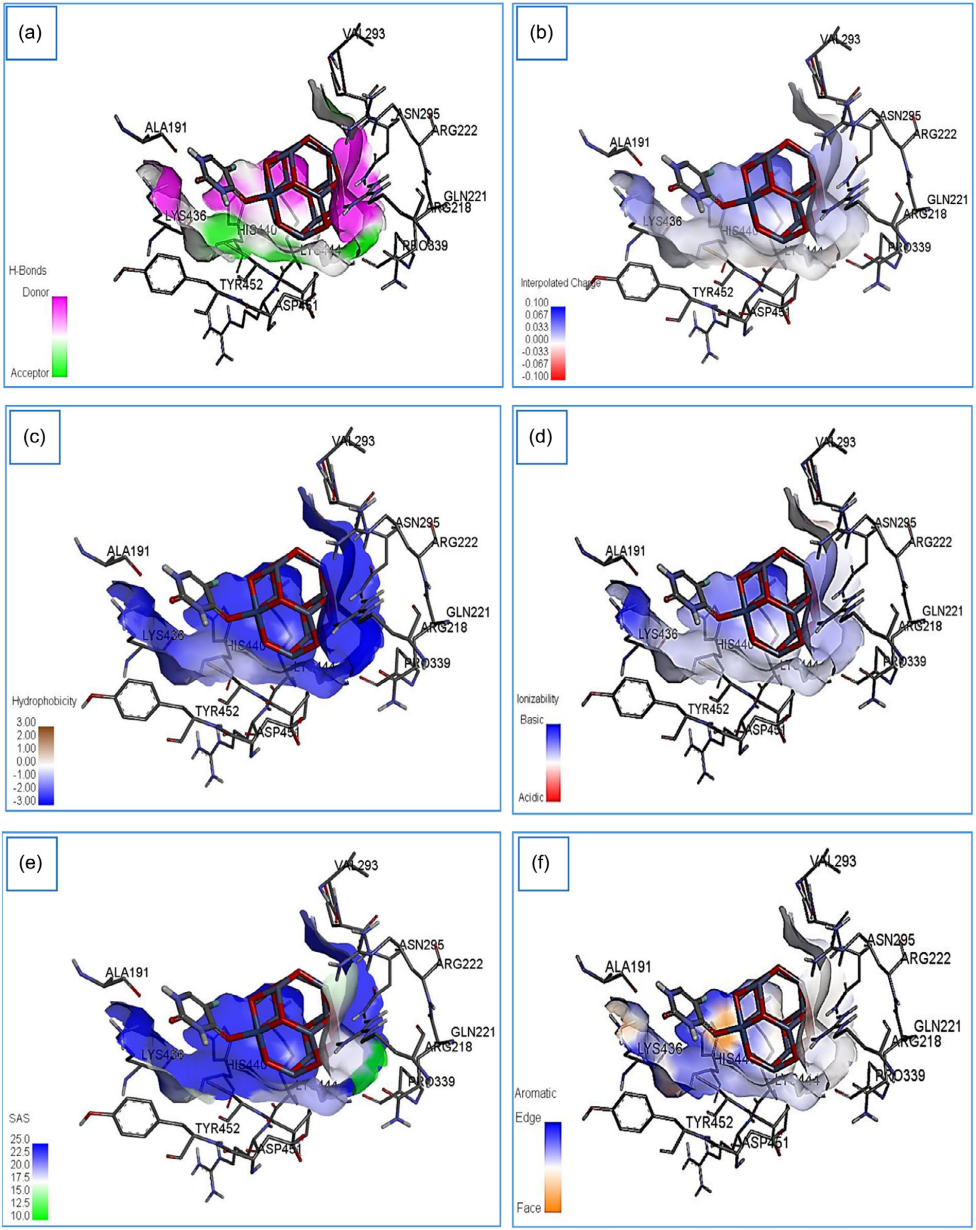
Nature of the HSA receptor surface in the 5‐FU@NiZn_11_O_12_ binding pocket showing the distribution of (a) hydrogen bonding, (b) interpolated charge, (c) hydrophobicity, (d) ionizability, (e) solvent‐accessible surface, and (f) aromatic edge and face.

A visual representation of docking is provided in Figure 10, where the flat ribbon model (Figure 10a) illustrates the spatial orientation of HSA with 5‐FU@NiZn_11_O_12_ nanocomplex embedded within its binding pocket. The zoomed‐in view (Figure 10b) highlights the key amino acid residues: Asp451, Pro447, His440, Asn295, Arg218, Cys448, Lys436, Glu294, Val293, and Tyr452 that form a network of interactions stabilizing the nanocomplex, reflecting a specific and stable binding orientation. Notably, the same binding site of HSA has been reported in previous studies [58, 59], underscoring the critical role of the active amino acids in molecular recognition and complex stabilization. Figure 11a–f further illustrates that the 5‐FU@NiZn_11_O_12_ nanocomplex binds to HSA through a combination of hydrogen bonding, electrostatic, polar, ionic, and aromatic interactions, resulting in a stable and favorable binding configuration.

Since 5‐FU exhibits weak binding affinity to HSA [17], this may result in a significant fraction of free drug in circulation and increase the risk of dose‐related side effects and drug–drug interactions, limiting its therapeutic precision [60]. The increased binding affinity of 5‐FU@NiZn_11_O_12_ nanocomplex to HSA may reduce the free 5FU concentration and the related side effects. Though the ZnO nanocage has been reported as a drug carrier, ZnO nanoparticles (NPs) could exhibit toxic effects due to their ability to generate reactive oxygen species (ROS), leading to oxidative stress and cellular damage [61, 62]. Binding of ZnO NPs to serum albumins (HSA/BSA) can partially mitigate this toxicity by shielding cells from direct exposure; however, such interactions are often accompanied by structural alterations in albumin, which may compromise its biological function [16, 63]. A study by Xia et al. [64] demonstrated that Fe‐doping effectively reduces the dissolution and subsequent toxicity of ZnO NPs in cells, zebrafish embryos, and rodent lungs. Similarly, Das et al. [65] reported that Cu‐doping of ZnO NPs increases cytotoxicity in a dose‐dependent manner by enhancing ROS generation and apoptosis through changes in electrical properties and cellular uptake. In addition, Yin et al. [66] reported that Fe‐doping of ZnO NPs lowered Zn ion release and cytotoxicity, whereas Mn‐doping increased oxidative stress and toxicity, and Co‐doping exhibited minimal cytotoxicity while maintaining strong antibacterial activity. Considering these challenges, we selected the interaction of 5‐FU with Ni‐doped ZnO nanocages using HSA as a carrier system, which may enhance the therapeutic action of 5‐FU.

The present DFT calculations are based on a finite nanocluster model and a specific exchange–correlation functional, which may not fully capture long‐range environmental or surface effects. Consequently, the computed energies and electronic properties are intended to describe relative trends rather than absolute experimental values. Similarly, molecular docking was performed using a rigid docking approximation, in which protein flexibility was not considered. As a result, the reported binding energies and inhibition constants should be interpreted qualitatively, providing comparative insight into binding affinity rather than exact thermodynamic values. In this context, molecular dynamics simulations could offer further dynamic insight into protein–nanocomplex interactions.

## 4 Conclusion

A comprehensive theoretical investigation of the structural, adsorption, electronic, and HSA interaction properties of fluorouracil (5‐FU) on TM‐doped ZnO (XZn_**11**_O_**12**_, where X = Zn, Cu, Fe, Ni) has been performed using DFT and molecular docking simulations. The results demonstrated that TM doping significantly enhanced the chemical reactivity and charge transfer activity of 5‐FU@Zn_**12**_O_**12**_ nanoclusters compared to the pristine structure, as evidenced by the reduced HOMO–LUMO gaps, particularly for the Ni–doped complex. Moderate values of adsorption energy (−20.97 kcal/mol), HOMO‐LUMO gap (2.44 eV), and binding energy with HSA (−5.36 kcal/mol) signify that 5‐FU@NiZn_11_O_12_ offers structural stability for drug sensing, delivery, and release. This study provides a solid theoretical foundation for the design of advanced nanocarriers in anticancer therapies. Further experimental studies are required to validate these findings and assess the biocompatibility and stability of the nanoclusters under physiological conditions.

## Author Contributions

**Mohan Bahadur Kshetri:** Experiments, data analysis, manuscript writing. **Navin Sharma:** Data analysis and manuscript writing. **Kamal Khanal:** Experiments, data analysis. **Madhav Prasad Ghimire:** Supervision and manuscript revision. **Tika Ram Lamichhane:** Conceptualization, supervision, and manuscript revision.

## Funding

This study was supported by Tribhuvan University (REF‐2081).

## Conflicts of Interest

The authors declare no conflicts of interest.

## Data Availability

The data supporting this study's findings are provided within the article.
